# Effects of Egg Yolk-Derived Peptide on Osteogenic Gene Expression and MAPK Activation

**DOI:** 10.3390/molecules190912909

**Published:** 2014-08-25

**Authors:** Hye Kyung Kim, Myung-Gyou Kim, Kang-Hyun Leem

**Affiliations:** 1 Department of Food & Biotechnology, Hanseo University, Seosan, Chungnam 356-706, Korea; E-Mail: hkkim111@dreamwiz.com; 2 College of Korean Medicine, Semyung University, Chungbuk 390-711, Korea; E-Mail: hkkim111@hanseo.ac.kr

**Keywords:** egg yolk peptide, osteogenic gene, MAPK, MG-63 cell

## Abstract

The present study investigated the effects of egg yolk-derived peptide (YPEP) on osteogenic activities and MAPK-regulation of osteogenic gene expressions. The effects of YPEP on cell proliferation, alkaline phosphatase activity, collagen synthesis, and mineralization were measured in human osteoblastic MG-63 cells. Activation of MAPKs and downstream transcription factors such as extracellular-signal-regulated kinase 1/2 (ERK1/2), c-Jun N-terminal kinase 1/2 (JNK1/2), p38, ELK1, and cJUN were examined using western blot analysis. YPEP dose-dependently increased MG-63 cell proliferation, ALP activity, collagen synthesis, and calcium deposition. YPEP activated ERK1/2, p38, and ELK1 phosphorylation whereas JNK and cJUN were not affected by YPEP. The *COL1A1* (collagen, type I, alpha 1), *ALPL* (alkaline phosphatase), and *SPP1* (secreted phosphoprotein 1, osteopontin) gene expressions were increased while *BGLAP* (osteocalcin) was not affected by YPEP. The ERK1/2 inhibitor (PD98509) blocked the YPEP-induced *COL1A1* and *ALPL* gene expressions as well as ELK1 phosphorylation. The p38 inhibitor (SB203580) blocked YPEP-induced *COL1A1* and *ALPL* gene expressions. SPP1 gene expression was not affected by these MAPK inhibitors. In conclusion, YPEP treatment stimulates the osteogenic differentiation via the MAPK/ELK1 signaling pathway. These results could provide a mechanistic explanation for the bone-strengthening effects of YPEP.

## 1. Introduction

Eggs have long been an important contributor to the nutritional quality of the human diet, and recognized as a very valuable source of proteins for human nutrition. Egg yolk is composed of various chemical substances that are important for human health [1]. We have previously reported that egg yolk water-soluble protein (YSP) stimulates proliferation, differentiation, and mineralization of osteoblasts [2]. Moreover, YSP exhibited anti-osteoporotic activity through increasing bone mineral density and prevent the cancellous bone loss in ovariectomized rat [3].

New advances in protein bioengineering are helping to explore numerous opportunities for releasing biologically functional peptides using specific enzymes. The resultant peptide could show enhanced or biologically new function with improved stability and/or solubility. Therefore, food scientists have paid a great deal of attention to peptides from partial enzymatic hydrolysates of food proteins as potential nutraceuticals and functional foods. Many biological peptides with health benefits including immune defense, uptake of nutrients, antihypertensive effect, and antibacterial activity have been identified from food protein hydrolysates [4,5,6].

Postmenopausal osteoporosis is characterized by low bone density which leads to increased bone fragility and greater susceptibility to fractures [7]. Current treatments for osteoporosis are dominated by drugs that inhibit bone resorption although they also suppress bone formation that may contribute to the pathogenesis of osteonecrosis [8]. To restore the extensive bone loss, there is a great need for anabolic treatments that induce osteoblasts to build new bone. Pre-osteoblastic cells produce proteins of the extra-cellular matrix, including type I collagen at first, and then to successively produce alkaline phosphatase (ALP) and osteocalcin during differentiation to osteoblasts [9]. Finally, osteoblasts deposit calcium.

Osteoblast maturation and differentiation can be modulated through many kinds of environmental factors and signaling cascades [10,11]. Many previous studies have shown that the expression of osteoblastogenic genes and functions are stimulated by mitogen-activated protein kinase (MAPK) signaling. For example, active form of extracellular-signal-regulated kinase (ERK) promotes osteoblast differentiation both *in vitro* and *in vivo* [12,13], activation of p38 has also been reported to be involved in osteoblastic differentiation by regulating the expression of ALP [14], and inactivation of c-Jun N-terminal kinase (JNK) significantly inhibited late-stage molecular events of osteoblast differentiation [15].

A variety of transcription factors and downstream kinases serve as substrates for activated MAPKs [16]. ELK1 is one of the major nuclear substrates of activated MAP kinases [16]. Upon their activation, MAPKs translocate to the nucleus where one of their major targets is the ELK1 [16]. Activated ERK1/2 and p38 phosphorylate many substrates, including ELK1 [17], leading to increased *c-fos* transcriptional activity. JNK phosphorylates c-Jun and also acts on ELK1 [18]. The phosphorylation of ELK1 transforms this protein into a potent trans-activator of transcription, thus the MAPK/ELK1 cassette plays a major role in signaling-driven gene activation [19].

In the present study, egg yolk-derived peptide (YPEP) was prepared, and its effects on proliferation, differentiation and mineralization of human osteoblastic MG-63 cells were explored. Furthermore, the effects of YPEP on MAPK activation and osteogenic gene expression were also explored. For the first time, we demonstrate that YPEP promotes the mRNA expressions of osteogenic genes through a MAPK/ELK1 signaling pathway.

## 2. Results and Discussion

### 2.1. YPEP Characteristics

The average size of the YPEP was between 0.7 and 1.4 kDa, with a mean molecular weight of 0.9 kDa, as determined by HPLC [20]. About 85% of the YPEP had a molecular weight of less than 3 kDa, and about 70% less than 1 kDa [20].

### 2.2. YPEP Enhances Osteogenesis

**Figure 1:**
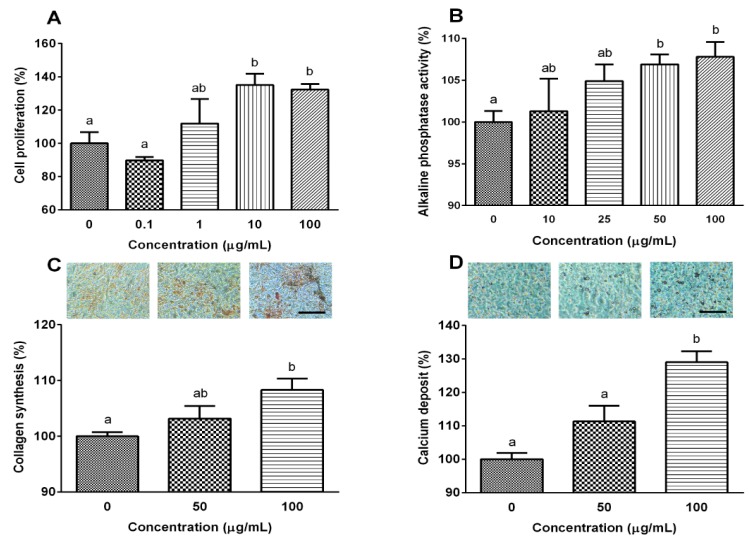
(**A**) Effects of YPEP on cell proliferation; (**B**) alkaline phosphatase activity; (**C**) collagen synthesis, and (**D**) calcium deposit. Data are expressed as a percentage of the control value, means ± SD of the three cultures. Values not sharing a common alphabet as superscripts are significantly different from each other at the level of *p* < 0.05.

Bone regeneration is regulated by a fine balance of biochemical and cellular events that ultimately stimulate osteoblasts to produce new tissue, in particular, new extracellular matrix composed mainly of collagen. The collagen matrix is then mineralized via ALP activity, which induces formation of calcium phosphate crystal seeds. Therefore, the effect of YPEP on osteoblast proliferation by BrdU incorporation was evaluated. The water soluble yolk peptide exhibited highest effect on osteoblast proliferation compared with whole egg peptide or egg whit peptide [20]. Therefore, water soluble yolk peptide was used in further study. As shown in Figure 1A, MG-63 cell proliferation was dose-dependently increased up to 10 μg/mL exhibiting 1.35-fold of the basal value when cells were treated with 10 μg/mL YPEP (*p* < 0.05). Next, the effects of YPEP on the differentiation of MG-63 cells were examined by determining ALP activity, collagen synthesis, and mineralization. YPEP treatment dose-dependently and significantly increased the ALP activity, an early-stage osteoblasts differentiation marker, exhibiting 6.9%, 7.2%, and 7.8% increase with 25, 50, and 100 μg/mL YPEP treatment, respectively (*p* < 0.05, Figure 1B). Collagen synthesis (Figure 1C) and calcium deposition (Figure 1D) assessed by Picro-Sirius Red and Alizarin Red-S stain, respectively, were also significantly increased by YPEP treatment compared with control group in a dose-dependent manner.

### 2.3. YPEP Activates MAPKs and ELK Pathway

Numerous growth factors, hormones, and cytokines have been shown to activate MAPKs in osteoblasts to induce cell proliferation and differentiation [16,21,22]. Therefore, in order to elucidate the mechanism underlying the osteogenic effects of YPEP, the activation of three different MAPKs (ERK1/2, JNK1/2, and p38) and its downstream proteins (JUN and ELK1) were examined using western blot analysis.

**Figure 2:**
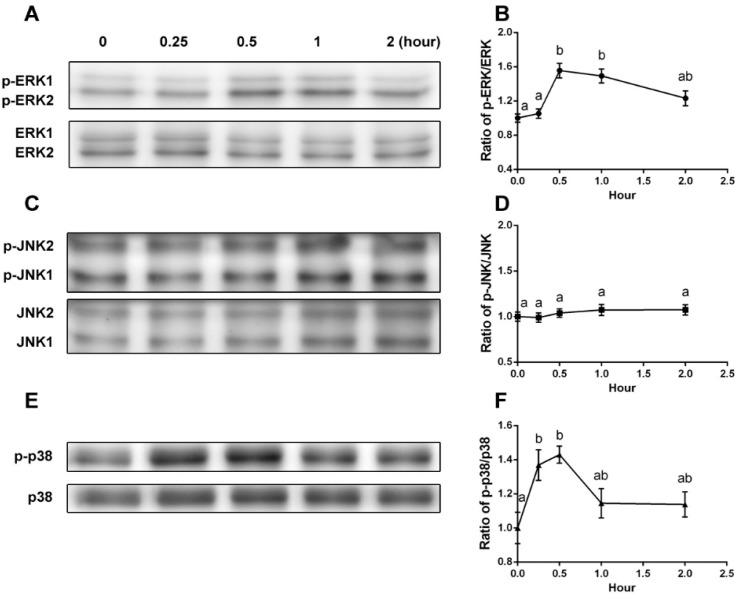
(**A**,**C**,**E**) Effects of YPEP on phosphorylations of ERK1/2, JNK1/2, and p38, respectively. (**B**,**D**,**F**) Densitometric results of ERK1/2, JNK1/2, and p38, respectively. Data are expressed as means ± SD of the three cultures. Values not sharing a common alphabet as superscripts are significantly different from each other at the level of *p* < 0.05.

YPEP (100 μg/mL) caused activation of ERK1/2 and p38 MAPK from 0.25 h to 0.5 h, and the activities returned to control levels at 2 h (Figure 2). The peak values of phosphorylated ERK1/2 and p38 at 0.5 h were 1.55 ± 0.09 fold of control for ERK1/2 and 1.43 ± 0.05 fold of control for p38 (Figure 2A,B,E,F). On the other hand, expressions of ERK1/2 and p38 (unphosphorylated form) were not altered by YPEP treatment. Also, YPEP failed to affect JNK1/2 activation (Figure 2C,D).

**Figure 3:**
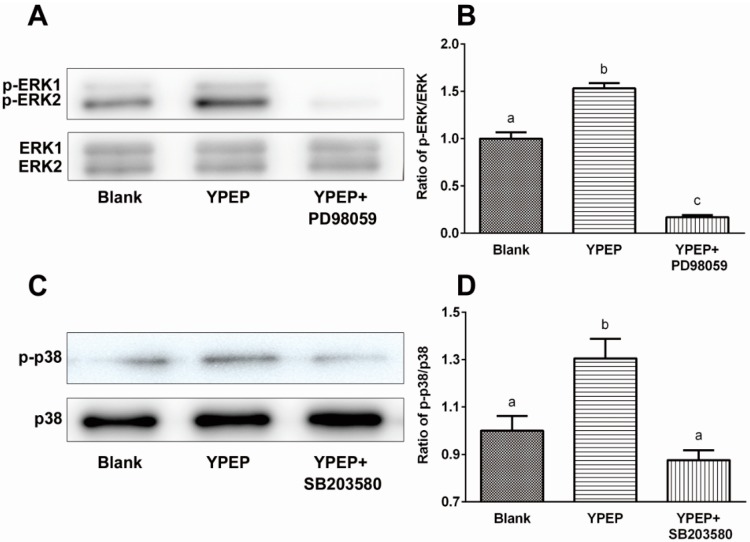
(**A**,**C**) Effects of MAPK inhibitor on YPEP-induced activations of ERK1/2 and p38; (**B**,**D**) Densitometric results of ERK1/2 and p38 phosphorylations. Data are expressed as means ± SD of the three cultures. Values not sharing a common alphabet as superscripts are significantly different from each other at the level of *p* < 0.05.

YPEP-mediated activations of ERK1/2 and p38 were additionally confirmed by each selective MAPK inhibitor to antagonize the effect of YPEP. YPEP treatment (100 μg/mL) significantly increased the ratios of phosporylated forms of ERK1/2 and p38 (1.53- and 1.30-fold, respectively, compared with control), and these YPEP-induced phosphorylations were completely abolished by inhibitors of ERK (PD98059) and p38 (SB203580) (Figure 3).

**Figure 4:**
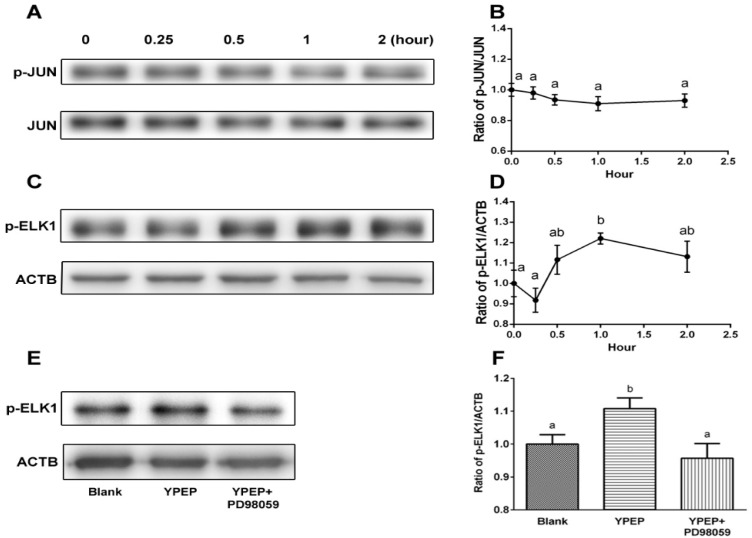
(**A**,**C**) Effects of YPEP on JUN and ELK1 activation. Western blot analysis of the JUN and ELK1; (**B**,**D**) Densitometric results of JUN and ELK phosphorylation; (**E**) YPEP-induced ELK phosphorylation was abolished by PD98059 (inhibitor of ERK1/2); (**F**) Densitometric result of ELK phosphorylation. ACTB: gene name of beta actin. Data are expressed as means ± SD of the three cultures. Values not sharing a common alphabet as superscripts are significantly different from each other at the level of *p* < 0.05.

We further examined the downstream proteins of MAPKs, ELK1 and JUN, because they have been shown to be a convergence point for MAPK cascades. Phosphorylated ELK1 protein expression was significantly increased by 1.22 ± 0.07 fold after 1 h of YPEP treatment (*p* < 0.05, Figure 4C,D). However, phosphorylation of JUN was not significantly affected by YPEP treatment (Figure 4A,B).

To elucidate the roles played by ERK/MAPK cascades in ELK1 phosphorylation, the cells were pre-treated with ERK inhibitor, PD98059, to inhibit the activation of MAPK-ERK1/2. PD98059 significantly reversed the YPEP-induced phosphorylation of ELK1 indicating that the effect of YPEP on ELK1 phosphorylation is ERK1/2 dependent (Figure 4E,F).

### 2.4. YPEP Enhances Osteogenic Gene Expression

**Figure 5:**
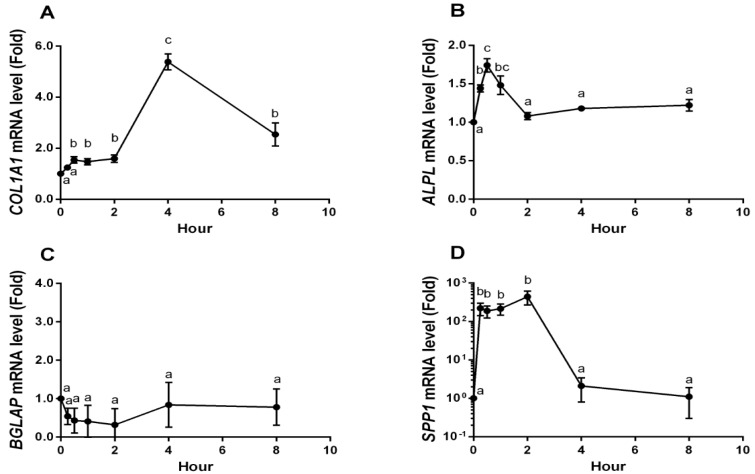
Effects of short term YPEP treatment on osteogenic gene expressions. (**A**) Collagen, type I, alpha 1 (*COL1A1*); (**B**) Alkaline phosphatase (*ALPL*); (**C**) Bone gamma-carboxyglutamic acid protein (*BGLAP*, osteocalcin); (**D**) Secreted phosphoprotein 1 (*SPP1*, osteopontin). Real-time PCR was used to measure mRNA levels at various time points following YPEP-treatment. Expression of *GAPDH* was used to normalize all samples. All data are expressed as mean ± SD of the three cultures. Values not sharing a common alphabet as superscripts are significantly different from each other at the level of *p* < 0.05.

During the bone formation process, there is increased expression of specific genes in osteoblasts and these genes play roles in extra cellular matrix formation and mineral deposition. To determine the YPEP-mediated regulation of the MAPK signaling in modulating osteogenic expression, MG-63 cells were differentiated in the presence of YPEP with or without specific ERK1/2 and p38 inhibitors, PD98059 and SB203580, respectively, and the expressions of osteogenic marker genes were analyzed for short term (8 h) and long term (7 days) period. The mRNA expressions of *COL1A1* (collagen, type 1, alpha 1), *ALPL* (alkaline phosphatase), *BGLAP* (bone gamma-carboxyglutamic acid protein, osteocalcin), and *SPP1* (secreted phosphoprotein 1, osteopontin) in MG-63 cells were measured by real-time PCR (Figure 5). Short term YPEP treatment caused transient and dramatic up-regulation of *COL1A1*, *ALPL*, and *SPP1* mRNA levels, whereas *BGLAP* expression was not affected by YPEP.

The *COL1A1* mRNA level was about 1.5-fold increased within 30 min after YPEP treatment, persisted for 2 h, and dramatically increased to 5.4-fold after 4 h (*p* < 0.05, Figure 5A). *ALPL* mRNA level was significantly increased within 30 min and decreased to basal level 2 h after YPEP treatment (*p* < 0.05, Figure 5B). The exponential notation of *SPP1* mRNA levels was significantly increased within 15 min, persisted for 2 h after YPEP treatment, and decreased to basal level (*p* < 0.05, Figure 5D). In contrast, *BGLAP* mRNA was not significantly affected by YPEP treatment (Figure 5C).

**Figure 6:**
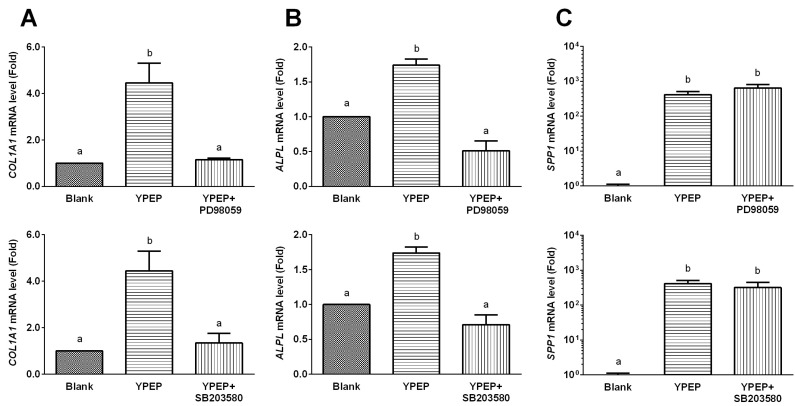
(**A**–**C** 1st row) Effects of ERK inhibitor (PD98059) on *COL1A1* (collagen, type I, alpha 1), *ALPL* (alkaline phosphatise), and *SPP1* (secreted phosphoprotein 1, osteopontin) gene expressions. (**A**–**C** 2nd row) Effects of p38 inhibitor (SB203580) on *COL1A1*, *ALPL*, and *SPP1* gene expressions. All data are expressed as mean ± SD of the three cultures. Values not sharing a common alphabet as superscripts are significantly different from each other at the level of *p* < 0.05.

Given that short term YPEP treatment induced both osteogenic gene expression and MAPK activation, we further explored the involvement of MAPK signaling pathway in YPEP-induced osteogenic gene expression using selective inhibitors of each MAPKs. The YPEP-induced *COL1A1* and *ALPL* mRNA gene expressions were completely blocked by specific inhibitors of ERK1/2 (PD98059) and p38 (SB203580) (Figure 6A,B). However, YPEP-induced *SPP1* mRNA gene expression was not affected by PD98059 and SB203580 (Figure 6C).

**Figure 7:**
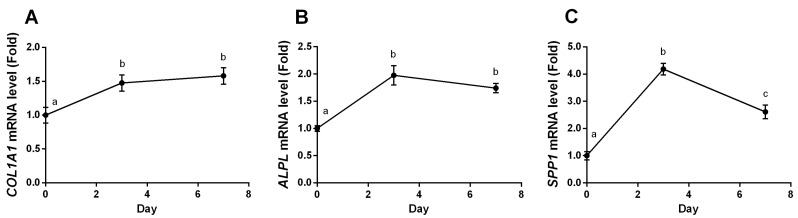
Effects of long term YPEP treatment on osteogenic gene expressions. (**A**) Collagen, type I, alpha 1 (*COL1A1*); (**B**) Alkaline phosphatase (*ALPL*); (**C**) Secreted phosphoprotein 1 (*SPP1*, osteopontin). All data are expressed as mean ± SD of the three cultures. Values not sharing a common alphabet as superscripts are significantly different from each other at the level of *p* < 0.05.

The osteogenic gene expressions in long term stage were determined for 7 days of YPEP treatment with or without MAPK inhibitors. The abolished effects of MAPK inhibitors for 7 days could not be determined because they reduced the cell viabilities to 50%–60% after 48 h treatment. The *COL1A1* and *ALPL* mRNA levels were 1.5-fold and 2.0-fold increased, respectively, after 3 days of YPEP treatment and persisted for 7 days (*p* < 0.05, Figure 7A,B). The *SPP1* mRNA level was about 4-fold increased on the 3rd day and decreased to 2.7-fold level on the 7th day of YPEP treatment (*p* < 0.05, Figure 7C).

### 2.5. Discussion

The present study demonstrated for the first time that YPEP (0.6~1.4 kDa) promoted osteoblast differentiation at least via ERK1/2 and p38/MAPK signaling pathway-mediated ALP action and collagen synthesis. Egg yolk protein has been reported to exhibit a positive effect on bone by increasing bone mineral density, bone mass content, and reducing bone loss in osteopenic bone [2,3]. In this study, YPEP stimulated the proliferation of the MG-63 cells in a dose-dependent manner, and the differentiation into osteoblasts represented by ALP activity and collagen deposition was also dose-dependently promoted by YPEP treatment. Furthermore, present study demonstrated the efficacy of YPEP as an anabolic agent for osteogenesis that can potentially up-regulate gene expressions responsible for bone formation such as *ALPL*, *COL1A1*, and *SPP1*. Type 1 collagen is an early marker of pre-osteoblast lineage which progressively expresses ALP during maturation stage and osteopontin/osteocalcin during mineralization phase [23]. Osteopontin appears prior to osteocalcin and expressed during the stage of active proliferation, and osteocalcin, secreted in the late stage of maturation, appears with mineralization [23]. Therefore, these data suggest that the YPEP may play role at early stage of osteoblast differentiation, and YPEP-induced *COL1A1*, *ALPL*, and *SPP1* gene expression could accelerate mineralization by hastening mineralization initiation and subsequently leading to an increase in the extent of calcium deposition.

**Figure 8:**
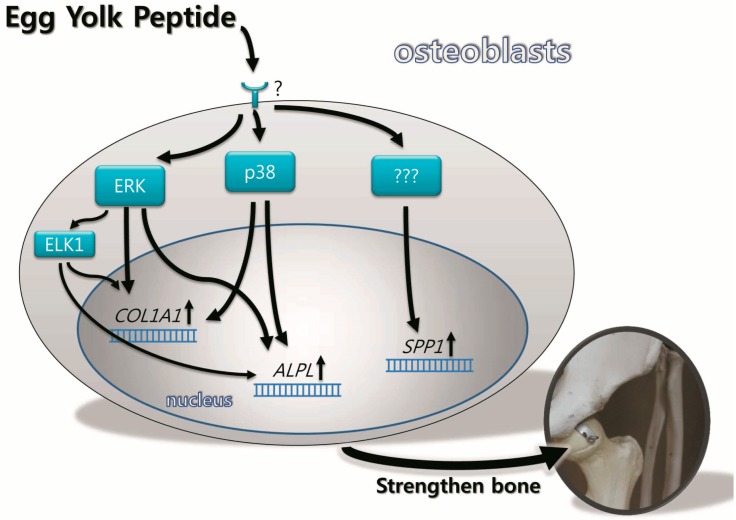
Schematic summary of the possible molecular mechanisms by which YPEP regulates the expression levels of COL1A1, ALPL, and SPP1.

Many previous studies have shown that the expression of osteoblastogenic genes and functions are stimulated by MAPK signaling, but its role remained debatable [12,13,14,15]. Lai *et al.* suggested that ERKs are essential for the growth and differentiation of osteoblasts [13], whereas Nakayama *et al.* reported that activation of ERK by growth factor stimulate MC3T3-E1 cell proliferation but suppress differentiation [24]. Suzuki *et al.* suggested that activation of p38 is critical for ALP expression in MC3T3-E1 cell induced by fetal calf serum [14]. Lately, a study using MC3T3-E1, mouse primary calvarial osteoblasts, and BMSC indicates that p38 MAPK, but not ERKs, is necessary for osteoblast differentiation [25]. Furthermore, ERK1/2 was reported to be involved in the ascorbic acid-induced *BGLAP* expression [26], and both ERK and p38 inhibitors completely abolished oscillatory flow-induced SPP1 mRNA level [27]. These ERK and p38-dependent *BGLAP* and *SPP1* expressions implied its regulatory function in the late phase of osteoblast differentiation. However, the results of the present study suggest that YPEP can activate ERK1/2 and p38 MAPK pathway at an early time point (Figure 2), and this is in accordance with elevated levels of gene expressions essential for the differentiation and mineralization process (*COL1A1*, *ALPL*, and *SPP1*). The regulation of MAPK on osteogenic gene expression was confirmed using MAPK inhibitors. YPEP-induced *COL1A1* and *ALPL* mRNA expressions were abolished by both ERK1/2 and p38 inhibitors suggesting that YPEP stimulated osteogenic differentiation via ERK1/2 and p38 MAPK signaling pathways (Figure 8). The YPEP-induced SPP1 mRNA expression was not affected by ERK1/2 or p38 inhibitors suggesting that these are not necessary elements for SPP1 mRNA expression.

The MAPK/ERK pathway is a chain of proteins in the cell that communicates a signal from a receptor on the surface of the cell to the DNA in the nucleus of the cell. G-protein-coupled receptors (GPCRs) are a large group of integral membrane receptors that transmit signals from a diverse array of external stimuli. Key component in GPCR-induced intracellular signaling is MAPK cascades [28]. Also, growth factors stimulate the ERK cascade by an elaborate mechanism initiated by receptor tyrosine kinase dimerization and autophosphorylation [28]. Therefore, GPCR and/or growth factor related receptors could be the candidates of YPEP-induced MAPK activation.

The biological effects of MAPKs are mediated by phosphorylation of downstream substrate and eventual activation of nuclear transcription factors. Therefore, the cis-elements in the promoters of COL1 and SPP1 were examined to identify the potential trans-acting factors that serve as the targets for ERK1/2 and JNK1/2 activations. Among the potential factors, ELK1 and JUN were chosen because they have been shown to be involved in the COL1 and SPP1 expression regulated by TGF-β, TNF-α, and other activated steroid hormone receptors [29,30]. In the current study, YPEP treatment increased the phosphorylation of ELK1 while there was no significant effect on JUN activation (Figure 4). Phosphorylation of EsLK1 was abolished by ERK1/2 inhibitor, suggesting ELK1 is a mediator of YPEP-induced up regulation of osteogenic gene expressions in MG-63 cells and ERK1/2 activity can be linked to the YPEP-induced phosphorylation of ELK1. Previous study have shown that the activation of ELK1 is involved in the expression of ALP and ostepontin mRNA in cementoblast cells [31]. The downstream target of ELK1 is the serum response element (SRE) of the c-FOS proto-oncogene [32,33], and over expression of c-FOS is considered as an elevated osteogenic activity [34].

Previously, egg yolk soluble protein was demonstrated to increase bone morphogenetic protein 2 (BMP2) in growing rats [35]. BMP2 plays important roles in cell growth and differentiation to bone development [36], and exhibits its osteogenic action by regulating the transcription factor including Runt-related transcription factor 2 (Runx2) [37]. Runx2 regulates the expression of several osteoblastic genes, including ALP, type I collagen, bone sialoprotein, osteocalcin, osteopontin and finally, mineralization of bone nodules [37]. Given the stimulatory effects of YPEP on osteoblast proliferation and early genes involved in osteoblast differentiation, further research is needed to identify the involvement of Runx2 in YPEP mediated action.

## 3. Experimental Section

### 3.1. Preparation and Characteristics of Egg Yolk Water-Soluble Peptide (YPEP)

Egg yolks were separated from hen eggs, homogenized and extracted with ethanol for preparation of yolk protein (YP). The YP fraction was filtered and fractionated for water solubility to obtain yolk water-soluble protein (YSP) as previously described [34]. YPEP was obtained by digestion of YSP with alkaline protease at 60 °C for 3 h, and provided by Pharmafoods International Co. (Kyoto, Japan). The range and the average of the molecular weight of the YPEP were determined by gel permeation chromatography-HPLC (Agilent 1200 series, Santa Clara, CA, USA). YPEP was dissolved in water at a concentration of 10 mg/mL and diluted with 30% acetonitrile containing 0.1% trifluoroacetic acid. The sample was centrifuged, filtered, and injected into a HPLC system with a Superdex Peptide column (30% CH_3_CN/0.1% trifluoroacetic acid, 0.5 mL/min). Absorbance was measured at 215 nm.

### 3.2. Cell Culture

Human MG-63 osteoblast-like osteosarcoma cell line (21427, Korean Cell Line Bank, Korea) were maintained in Dulbecco’s Modified Eagle Medium (DMEM, Invitrogen, Grand Island, NE, USA) containing 10% FBS (heat-inactivated, Invitrogen) and 100 U/mL penicillin/streptomycin (Sigma-Aldrich, St. Louis, MO, USA) in an incubator with a humidified atmosphere of 95% air and 5% CO_2_ at 37 °C.

### 3.3. Cell Proliferation and Alkaline Phosphatase Activity

The effect of YPEP on the proliferation of MG-63 cell was determined by a colorimetric immunoassay kit (Roche Diagnostics, Basel, Switzerland), which is based on quantitating bromodeoxyuridine (BrdU) incorporation into the newly synthesized DNA. Briefly, MG-63 cells, incubated in DMEM, were seeded in 96-well plates (5000 cells/well) and incubated overnight. YPEP (0.1, 1, 10, and 100 μg/mL) were added to the cells and incubated for 24 h. Subsequently, BrdU was added to each well and reincubated for 2 h. After removing the culture medium, the cells were fixed and the DNA was denatured by incubated with FixDenat solution for 30 min. FixDenat solution was removed and incubated with anti-BrdU-POD solution for 90 min. The plate was developed by substrate solution and the absorbance was measured at 450 nm (BioTek Inc., Winooski, VT, USA). ALP was measured using enzymatic assay of alkaline phosphatase. MG-63 cells, incubated in DMEM, were seeded in 6-well plates (20,000 cells/well) and incubated overnight. YPEP (10, 25, 50, and 100 μg/mL) were added and reincubated for 24 h. Then the cells were washed with PBS and lysed in 300 μL of 0.2% of Triton X-100 by shaking for 20 min at room temperature. The cell lysate were centrifuged at 4 °C 13,000 rpm for 5 min. The supernatant of the lysate were used to measurement of alkaline phosphatase activity. 100 μL of sample were transferred into new 96 well plate and add 100 μL of 22.4 mM of *p*-nitrophenyl phosphate solution in 2 M of diethanolamine and 1 mM MgCl_2_ buffer (pH 9.8 at 37 °C). The plates were incubated at 37 °C and absorbance at 450 nm was measured.

### 3.4. Collagen Content

Collagen synthesis was measured using Picro-Sirius Red method [38]. MG-63 cells were seeded in 12-well plates (10,000 cells/well) and allowed to adhere overnight. The medium was changed to osteogenic medium (DMEM with 10 mM β-glycerophosphate, 5 nM dexamethasone, and 50 μg/mL ascorbic acid), YPEP was added to the cells and incubated for 7 days. Cells were fixed with Bouin’s fluid and stained with 0.1% Sirius red (Direct Red 80, Sigma-Aldrich) in a saturated aqueous solution of picric acid for 30 min. Each well was observed using an optical microscope, and stained dye was dissolved and the absorbance was measured at 540 nm (BioTek Inc., Winooski, VT, USA).

### 3.5. Calcium Deposition

The formation of calcium phosphate was determined by alizarin red-S assay [39]. MG-63 cells were seeded and incubated as described above in the collagen assay process. After 7 days, cells were fixed with 10% formaldehyde and stained with 2% of Alizarin Red-S (pH 4.2, Sigma-Aldrich) at room temperature. The stained alizarin red-S was extracted with 10% acetic acid, and the amount of calcium deposition was quantified using the absorbance of extracted alizarin red-S at 405 nm (BioTek Inc.).

### 3.6. Western Blot Analysis

Cells, pretreated with or without an inhibitor of ERK (PD98059) and p38 (SB203580) for 30 min, were incubated with YPEP (100 μg/mL) for various times (0–2 h). Proteins were extracted with PRO-PREP Protein Extraction solution (Intron Biotechnology, Seongnam, Korea). Aliquots of samples were subjected to 10% SDS-PAGE, and transferred onto a PVDF membrane. After blocking, the membrane was incubated with the primary antibody (anti-JNK, anti-p-JNK, anti-ERK, anti-p-ERK, anti-cJUN, anti-p-cJUN, anti-p-ELK-1, and anti-ACTB antibodies raised in rabbit, Cell Signaling Technology, Danvers, MA, USA) followed by incubation with anti-rabbit secondary antibody conjugated with horseradish peroxidase (Cell Signaling Technology). Immunoreactive bands were visualized using chemiluminescent imaging system (Fusion SL2, Vilber Lourmat, Marne-la-Vallée Cedex, France), and analyzed by the Bio1d software (Vilber Lourmat). 

### 3.7. Real-Time PCR

Cells were pretreated with or without an inhibitors of MAPKs and incubated with YPEP (100 μg/mL) for various time points (0–8 h). Total RNA was extracted using RNeasy^®^ Protect Mini kit (Qiagen, Valencia, CA, USA), and cDNA was synthesized from mRNA using QuantiTect^®^ Reverse Transcription kit (Qiagen). Real-time PCR was performed using QuantiTectTM SYBR^®^ Green PCR kit (Qiagen) according to the manufacturer’s protocol. The PCR primer sequences are shown in Table 1. Analyses were performed using Rotor-Gene Q^®^ (Qiagen) and gene expression values were calculated based on the comparative ΔΔ CT method [40].

**Table 1:** Oligonucleotide sequences of osteogenic genes.

Primer	Direction	Sequence
collagen, type I, alpha 1 (*COL1A1*)	Forward Reverse	5'-GCG GCT CCC CAT TTT TAT ACC-3' 5'-GCT CTC CTC CCA TGT TAA ATA GCA-3'
alkaline phosphatase (*ALPL*)	Forward Reverse	5'-AAA CCG AGA TAC AAG CAC TCC CAC-3' 5'-TCC GTC ACG TTG TTC CTG TTC AG-3'
osteocalcin (*BGLAP*)	Forward Reverse	5'-CCA TGA GAG CCC TCA CAC TCC TC-3' 5'-GCT TGG ACA CAA AGG CTG CAC-3'
osteopontin (*SPP1*)	Forward Reverse	5'-AGG CTG ATT CTG GAA GTT CTG AGG-3' 5'-GAC TTA CTT GGA AGG GYC TGT GGG-3'
housekeeping gene (*GAPDH*)	Forward Reverse	5'-TCA TCA ATG GAA ATC CCA TCA CC-3' 5'-TGG ACT CCA CGA CGT ACT CAG C-3'

### 3.8. Statistical Analysis

All experiments were performed with triplicate independent samples. Data are expressed as mean ± SD. All data sets were analyzed by One-way ANOVA followed by Tukey’s *post-hoc* test to determine statistical differences (GRAPHPAD prism ver. 6). *p* < 0.05 was considered significant.

## 4. Conclusions

The present preliminary study, focused on the MAPK signaling pathway, has shown that YPEP promotes osteogenic activities and up regulates bone matrix genes via the MAPK/ELK1 signaling pathway. YPEP-mediated *COL1A1* and *ALPL* gene expression requires ERK1/2 and p38 MAPK activation, while *SPP1* gene expression does not require MAPK activation. These results provide a mechanistic explanation for the osteogenic effects of YPEP in MG-63 human osteoblast-like cells.
